# Meniscus Matrix Structural and Biomechanical Evaluation: Age-Dependent Properties in a Swine Model

**DOI:** 10.3390/bioengineering9030117

**Published:** 2022-03-15

**Authors:** Lucia Aidos, Silvia Clotilde Modina, Valentina Rafaela Herrera Millar, Giuseppe Maria Peretti, Laura Mangiavini, Marco Ferroni, Federica Boschetti, Alessia Di Giancamillo

**Affiliations:** 1 Department of Biomedical Sciences for Health, Università degli Studi di Milano, 20133 Milano, Italy; lucia.aidos@unimi.it (L.A.); valentina.herrera@unimi.it (V.R.H.M.); giuseppe.peretti@unimi.it (G.M.P.); laura.mangiavini@unimi.it (L.M.); 2 Department of Veterinary Medicine and Animal Science, Università degli Studi di Milano, 26900 Lodi, Italy; silvia.modina@unimi.it; 3 IRCCS, Istituto Ortopedico Galeazzi, 20161 Milano, Italy; federica.boschetti@polimi.it; 4 Department of Chemistry, Material and Chemical Engineering “Giulio Natta”, Politecnico di Milano, 20133 Milano, Italy; marco.ferroni@polimi.it

**Keywords:** meniscus morphology, pig, biomechanics, matrix, traction, compression

## Abstract

The analysis of the morphological, structural, biochemical, and mechanical changes of the Extracellular Matrix (ECM), which occur during meniscus development, represents the goal of the present study. Medial fully developed menisci (FD, 9-month-old pigs), partially developed menisci (PD, 1-month-old piglets), and not developed menisci (ND, from stillbirths) were collected. Cellularity and glycosaminoglycans (GAGs) deposition were evaluated by ELISA, while Collagen 1 and aggrecan were investigated by immunohistochemistry and Western blot analyses in order to be compared to the biomechanical properties of traction and compression tensile forces, respectively. Cellularity decreased from ND to FD and GAGs showed the opposite trend (*p* < 0.01 both). Collagen 1 decreased from ND to FD, as well as the ability to resist to tensile traction forces (*p* < 0.01), while aggrecan showed the opposite trend, in accordance with the biomechanics: compression test showed that FD meniscus greatly resists to deformation (*p* < 0.01). This study demonstrated that in swine meniscus, clear morphological and biomechanical changes follow the meniscal maturation and specialization during growth, starting with an immature pattern (ND) to the mature organized meniscus of the FD, and they could be useful to understand the behavior of this structure in the light of its tissue bioengineering.

## 1. Introduction

The menisci are fibrocartilaginous structures interposed between the femoral condyles and tibial plateau of all mammals. They are fundamental for the knee balance, because the femoro-patellar-tibial joint is, basically, an imperfect hinge [1]. Furthermore, knee kinematics is characterized by a three-plan movement, that is, different moduli of solicitation develop in this joint [2]. Therefore, menisci have multiple functions in this joint: they act as shock absorbers, they can bear loads, and they allow joint stability, congruity, and lubrication [3]. If the stifle presents a structural abnormality or a previous or concomitant disease, the different load and stress distribution over the two menisci will lead to meniscal injuries and developing of osteoarthritis (OA) due to the failure of meniscal protective function in the joint [4]. For these reasons, the recognition of the clinical importance of the meniscus has led the development of more detailed studies regarding this particular anatomical structure [5,6] in the light of its biomechanical properties.

The meniscus is an inhomogeneous and anisotropic material because its proprieties vary non-linearly with the location and direction of stimuli, depending on the distribution and organization of proteoglycans and collagen [7]. Meniscal biomechanical properties depend on the complex structure and organization of the extracellular matrix (ECM). The main ECM meniscal components are water, collagen, and GAGs (principally aggrecan), and they work together to resist to the different forces that act on menisci. In adult pigs, collagen type I is arranged in circumferential bundles all along the inner part of meniscus and, co-expressed with collagen type II, in radial bundles [8]; this disposition of the fibers reflects the directions of the two principal forces that act on the meniscus: circumferential/tensile force and gait/load force. Aggrecan has a complex interaction with collagen fibrils. Thereby, aggrecan enables the tissue to resist compressive forces by locking itself within the collagen meshwork and absorbing large amounts of water from the surrounded environment, and it exerts a swelling pressure on the collagen meshwork [9]. Within ECM, collagen fibrils resist such swelling. If aggrecan and collagen are in equilibrium, the tensile forces that stretch the collagen fibrils balance the swelling of the aggrecan itself. During compression, this equilibrium is perturbed: compression displaces water and this increases the swelling potential of the aggrecan, while upon removal of the compression, the aggrecan will re-swell and restore the original equilibrium [10]. Therefore, the complex disposition of collagen fibers in the meniscus supplies its tensile properties, whereas aggrecan expresses important weight-bearing properties in this fibro-cartilaginous tissue [11].

Furthermore, a stress over meniscal tissue leads to a different response by fibrocartilage that is an intrinsic viscoelastic and an extrinsic poroelastic response. More precisely, under normal physiologic loading, the meniscus experiences high tensile and shear stresses as well as compressive stress [12], which can lead to injury. Unfortunately, the meniscus is characterized by a scarce vascularization [13,14,15,16], which is confined to its outer third in the adult meniscus; thus, its regeneration and ability to repair are extremely reduced, if not absent. Many therapeutic procedures for meniscal tears have been applied over the years [17]. None of these procedures, however, has the capacity of preventing the development of OA in the knee joint. Nowadays, the treatment goal is not just to remove the damaged part of the meniscus, but to rebuild it or eventually replace it. Of course, this idea is still far from its immediate realization, although many attempts have already been made [18,19,20]. For this reason, more studies are necessary to increase our knowledge about this small but essential structure of ECM. In the present study, we focused on the medial meniscus since 60–80% of the weight acts on the medial part of the knee joint [21]. Meniscal ECM composition, structure, and biomechanics make up basic fundamental information for engineering meniscal tissue substitutes. The goal of the present study is represented by the characterization of the ECM morphological, structural, biochemical, and mechanical properties of meniscus and their changes during growth.

## 2. Materials and Methods

**Figure 1:**
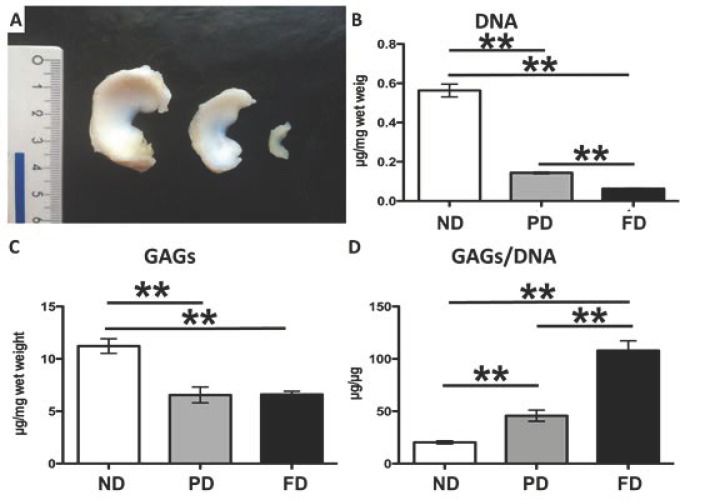
(**A**) Macroscopic aspect and comparison of menisci belonging to the three classes of age. (**B**–**D**) Differences in biochemical analyses of menisci belonging to the three classes of age: (**B**) DNA quantification; (**C**) GAGs quantification; (**D**) GAGs/DNA ratio. Quantification of DNA normalized to the wet weight (µg/mg). Values with ** differ for *p* < 0.01.

For the aim of this study, we obtained menisci from a local slaughterhouse, which breeds swine Landrace x Large White. Knees of adults (~9 months old, weight 130–140 kg), young (1 month old, weight 10–12 kg), and neonates (stillbirths or dead in peripartum, weight 1–1.5 kg) were collected (Figure 1A). From the morphological point of view, the meniscus is fully developed in 9-month-old pigs, partially developed in 1-month-old pigs, and not developed at birth [22]. We named the 9-month-old pigs FD (fully developed), the 1-month-old pigs PD (partially developed), and the newborn ND (not developed). The swine model was chosen because of its similarities with the human meniscus, its easy availability, and its wide use in the literature as a model for meniscal tissue engineering and repair [23,24,25,26]. The joints were dissected to isolate lateral and medial menisci. Capsular tissue and ligaments were gently removed, and the medial menisci were stored in saline solution NaCl 0.9% and refrigerated. No animal has been sacrificed for the purposes of this study. The Ethic Committee of the University of Milan (OPBA, 58/2016) approved the use of cadavers for this research purpose. All animals that were used were died for reasons that have no relationship with the present study. 

### 2.1. Biochemical Analyses

Medial meniscal samples were used for the biochemical analysis and Western blot (see below). Whole menisci of all animals (n = 8 ND, n = 8 PD, n = 8 FD, n = 24 total menisci) were treated as described elsewhere [6,16,27,28]. Briefly, the samples were digested in papain (Sigma-Aldrich, Milan, Italy) for 16–24 h at 60 °C: 125 µg/mL of papain in 100 mM sodium phosphate, 10 mM sodium EDTA (Sigma-Aldrich), 10 mM cysteine hydrochloride (Sigma-Aldrich), and 5 mM EDTA adjusted to pH 6.5 and brought to 100 mL of solution with distilled water. Later, the digested samples were assayed separately for proteoglycan and DNA contents. Proteoglycan content was estimated by quantifying the amount of sulphated glycosaminoglycans using the 1,9-dimethylmethylene blue dye binding assay (Polysciences, Inc., Warrington, PA, USA) and a microplate reader (wavelength: 540 nm). The standard curve for the analysis was generated by using bovine trachea chondroitin sulphate A (Sigma). DNA content was evaluated with the Quant-iT Picogreen dsDNA Assay Kit (Molecular Probes, Invitrogen Carlsbad, CA, USA) and a fluorescence microplate reader and standard fluorescein wavelengths (excitation 485 nm, emission 538 nm, cut-off 530 nm). The standard curve for the analysis was generated using the bacteriophage lambda DNA supplied with the kit.

### 2.2. Protein Extraction and Western Blot

For each experimental group (ND, PD, FD), 8 specimens were processed (total nr = 24). The samples were pulverized for 2 min at 3000 oscillations/min in a liquid nitrogen cooled dismembrator (Mikro-Dismembrator, Sartorius Stedim, Muggio, Italy). They were then homogenized in a buffer containing 50 mM TrisHCl, 150 mM NaCl, 0.1% SDS, 0.5% sodium deoxycholate, and 1% NP40, at pH 7.4, supplemented with protease inhibitor cocktail (Euroclone, Pero, Italy). They were centrifuged at 13,000× *g* at 4 °C for 10 min to discard cellular debris. The total protein concentration was determined using bicinchoninic acid assay (BCA) (Euroclone), which exhibits a color change of the sample solution from green to purple in proportion to protein concentration, and the color tone could then be measured. After addition of 0.05% bromophenol blue, 10% glycerol, and 2% β-mercaptoethanol, 50 µg of each sample was boiled and loaded onto 6% SDS–polyacrylamide gels. After gel electrophoresis, polypeptides were transferred to nitrocellulose filters (Sigma-Aldrich), and these membranes were incubated with 5% non-fat milk for 1 h at room temperature to block non-specific sites. After this, all samples were probed using the following antibodies (Abs): anti-collagen 1 (1:500; no. NB600–408; Novus Biologicals, Littleton, CO, USA), anti-aggrecan (1:1000; BC-3, ab3773, Abcam, Cambridge, CB2 0AX, UK), and anti-GAPDH (clone GAPDH-71.1; Sigma-Aldrich) and kept at room temperature for 2 h. The filters were then washed and incubated for 1 h always at room temperature with HRP-labeled secondary antibodies (1:5000; Bio-Rad, Hercules, CA, USA). For the last step of the procedure, the blots were developed using a chemiluminescent substrate (WESTAR Nova 2011, Cyanagen, Bologna, Italy) to detect and characterize the proteins C, as previously described. A loading control was used to normalize the data by measuring the levels of GAPDH (a marker for total protein in each sample) in order to compare target protein expression levels between ND, PD, and FD menisci.

### 2.3. Micro Anatomical Analysis: Immunohistochemistry

**Figure 2:**
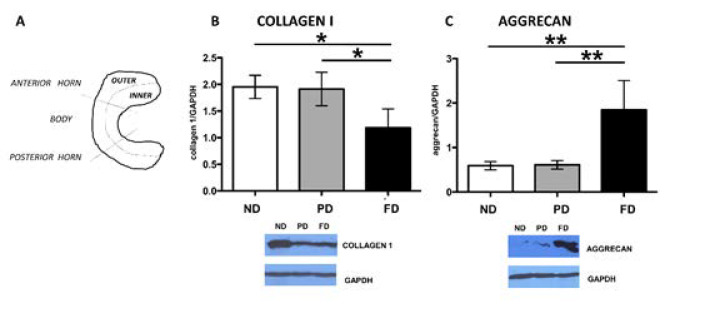
(**A**) Meniscal partition scheme: menisci were subdivided transversally (in an anterior horn, a body, and a posterior horn) and longitudinally, in an inner and an outer part; Western blot results for collagen type I (**B**) with GAPDH and subsequent calnexin as housekeeping; Western blot results for aggrecan (**C**) with GAPDH and subsequent calnexin as housekeeping. ND: not developed; PD partially developed; FD: fully developed. Values with ** and * differ for *p* < 0.01 and *p* < 0.05, respectively.

**Figure 3:**
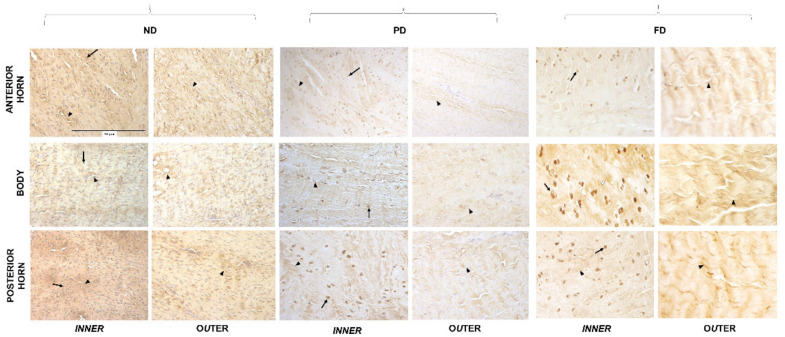
Collagen I immunostaining in the inner and outer regions of the anterior horn, body, and posterior horn of ND, PD, and FD. Arrows: nuclei, arrowheads: fibers. All the images have the same scale bar (located in the first image: 50 μm).

Medial menisci were analyzed (8 per group, n = 24) and they were divided, through two radial-transversal cuts, into three different parts: anterior horn, body, and posterior horn. Subsequently, each part was subdivided into an inner and an outer part through a longitudinal cut (Figure 2A and Figure 3). Samples were then fixed in 10% (*v/v*) phosphate-buffered formaldehyde (n = 24); dehydrated in a graded 50% (*v/v*), 70% (*v/v*), 95% (*v/v*), and 100% (*v/v*) ethanol series; embedded in paraffin; and cut into 4 µm thick consecutive sections. Each sample was used for two different IHC protocols. After rehydration, sections were washed in Phosphate Buffered Saline (PBS, pH 7.4) plus Triton for 5 min; moreover, block endogenous peroxidase using H_2_O_2_ for 8 min in a humidified chamber was applied, and subsequently the slides were incubated with the first-step primary antiserum, [1:50] collagen 1 (1:500; no. NB600–408; Novus Biologicals, Littleton, CO, USA) for 24 h at 18–20 °C in a humid chamber, then washed in PBS, and subsequently treated with EnVision anti-rabbit system (Dakocytomation, Milan, Italy). The sections were washed in PBS for 10 min and incubated with a 5 min 3,3’ diaminobenzidine tetrahydrochloride (DAB)/hydrogen peroxide, which results in a brown precipitate at the antigen site. Counterstaining with Mayer hematoxylin for 1 min allowed a better visualization of the morphological structure; then, the slides were dehydrated and permanently mounted. For the second IHC, all steps were identical to the abovementioned protocol, except for a few details: primary antibody was anti-aggrecan diluted in PBS at a concentration of 1:10 (ab3773, Abcam, Cambridge, MA, USA), and prior to antibody processing and antigen retrieval, 0.01 Units Chondroitinase (Sigma-Aldrich) in PBS for 10 min was applied. Secondary antibody was EnVision anti-mouse. The specificity tests for the antibodies were verified by incubating sections with: (i) PBS instead of the specific primary antibody; (ii) PBS instead of the secondary antibodies. The results of these controls were negative (i.e., staining was abolished). Photomicrographs were taken with an Olympus BX51 microscope (Olympus, Milan, Italy) equipped with a digital camera, and final magnifications were calculated.

### 2.4. Biomechanical Analysis

Menisci from FD, PD, and ND swine were stored in saline solution NaCl 0.9% and frozen at −80 °C until the time of testing (8 per group, n = 24) as described by Ferroni et al. [22]. At least 24 h before test execution, samples were taken to a temperature of −24 °C and then completely thawed at room temperature (23 °C). Each meniscus was prepared and cut in a different shape depending on the test run. Compression and circumferential traction tests were performed using EnduraTEC ELF^®^ 3200 machine (serial number 1–866–835–1800; 3200; Minnetonka, MN, USA), equipped with a 220 or 22 N load cell depending on the test and sample.

**Figure 4:**
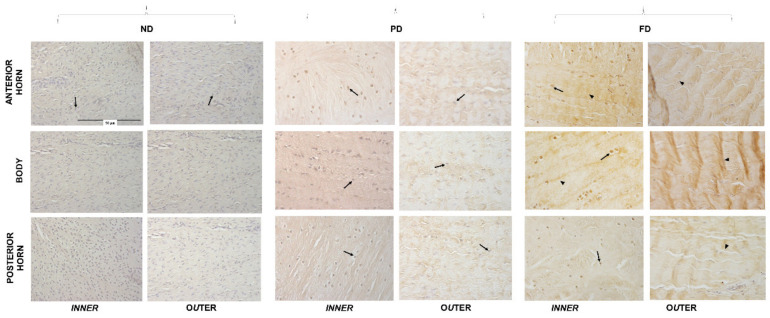
Aggrecan immunostaining in the inner and outer regions of the anterior horn, body, and posterior horn of ND, PD, and FD. Arrows: nuclei, arrowheads: fibers. All the images have the same scale bar (located in the first image: 50 µm).

Compression test—For compression tests, specimens were obtained dividing each meniscus in the three portions: anterior horn, body, and posterior horn. Subsequently, for each zone, a cylindrical part perpendicular to the femoral and tibial surfaces was cut using a die cutter (Figure 4). The diameter of the specimens was dependent on the original meniscus. Anterior horn, posterior horn, and body of ND menisci were analyzed as a whole, and no further divisions were made. Before testing, dimensional measurements on the specimens were made with a digital caliper (Mitutoyo Corp, Kanogawa, Japan, number of 06,081,911 series, accuracy class 1). The samples were inserted into a Plexiglas cell, and PBS solution was added into the cell to avoid dehydration of the specimen. The thickness of all samples was measured from the position of the testing machine actuator, after imposing a preload of approximately 0.01 N (ND and PD menisci) or 0.1 N (FD menisci). The sample was then subjected to a multi-ramp stress relaxation test, made of five increasing 4% strains at a velocity of 0.1%/s, followed by stress relaxation to equilibrium for 600 s (ND and FD menisci) or 2000 s (PD menisci). The compressive Young modulus, EC, was obtained for each ramp from the equilibrium data as the ratio between values of relaxation stress and the corresponding values of strain. 

**Figure 5:**
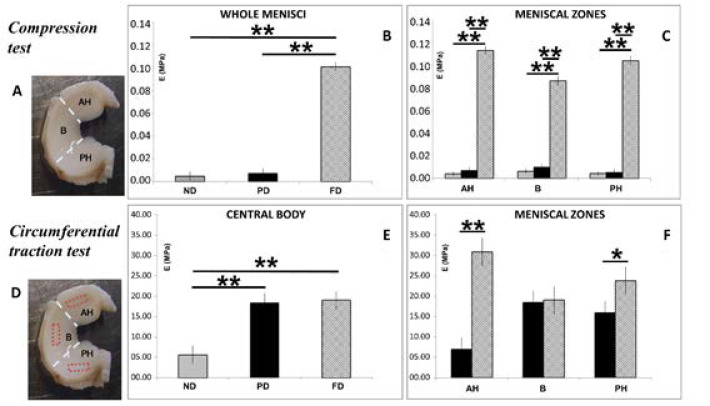
Biomechanical results from compression (**A**) and traction (**D**) tests. Compression test results for pooled (**B**) and subdivided samples (**C**). Circumferential traction test results for pooled (**E**) and subdivided (**F**) samples. AH: anterior horn; B: body; PH: posterior horn. Values with ** and * differ for *p* < 0.01 and *p* < 0.05, respectively.

Circumferential traction test—For this test, we obtained representative fragments of the meniscus anterior horn, body, and posterior horn. These fragments were cut out using a scalpel, trying to obtain samples with a shape as similar as possible to a parallelepiped (Figure 5D) and following the circumferential tensile force direction. Before testing, dimensional measurements on the specimens were made with the same digital caliper previously indicated. Width and thickness of each sample were obtained using the caliper, and length was measured on the mounted specimen, considering as length the distance between the two grips after preload application. A preload of approximately 0.01 N (PD and NT menisci) or 0.1 N (FD menisci) was applied. The ND menisci are too small to be divided into the three anatomical zones for the tensile test evaluation: in fact, only central body was analyzed because anterior and posterior horns were used to lock the specimen into the machine. The specimens were subjected to a multi-ramp stress relaxation test, made of four increasing 4% strains at a velocity of 0.1%/s, followed by stress relaxation to equilibrium for 1200 s. A custom-made chamber filled with PBS was used to keep the samples hydrated during the test. The tensile relaxation Young moduli, ET, were determined for each ramp from the equilibrium data as the ratio between the relaxation stress value and the corresponding value of strain.

### 2.5. Statistical Analyses

Statistical analysis of the data (biochemical and biomechanical results: ND, PD, FD, and all the comparisons) was performed using the general linear model of the SAS (version 8.1, Cary Inc., NC). The individual meniscal samples were considered to be the experimental unit of all response variables. The data were presented as least squared means ± S.E.M. Differences between means were considered significant at *p* < 0.05 and highly significant at *p* < 0.01.

## 3. Results

### 3.1. Biochemical Analyses

The biochemical analysis showed a significant decreasing cellularity from ND to PD and FD (*p* < 0.01 all comparisons, Figure 1B), while GAGs were revealed to be higher in ND vs. PD and FD vs. ND (*p* < 0.01, Figure 1C), and the GAGs/DNA ratio significantly increased with age from ND to PD and FD (*p* < 0.01 all comparisons, Figure 1D).

### 3.2. Western Blot

Collagen I showed a progressive decrease in the different age groups: ND and PD groups revealed a significantly higher level of collagen I if compared to the FD one (*p* < 0.05, Figure 2B). Conversely, aggrecan presented a statistically higher level in FD vs. the other groups, which were comparable (*p* < 0.01, Figure 2C).

### 3.3. Microanatomical Analysis: Immunohistochemistry

Collagen type I inner part—Collagen type I showed fibers (Figure 3, arrowhead) as well as nuclear immunopositivity (Figure 3, arrow) in the inner part of anterior horn, body, and posterior horn of all the different stages of maturation of the meniscus. ND samples showed a higher cellularity with weak nuclear positivity of fibroblasts, but no specific pattern of organization of the fibers in the three considered zones. In PD samples, a decreased cellularity was evident with nuclear positivity in fibroblast cells of the three zones: in the posterior horn, a mature cellular phenotype, i.e., fibro-chondrocytes, was evident (Figure 3, arrow). Fibers of collagen I with a linear trend were visible in the matrix (Figure 3, arrowhead). FD menisci presented an intense nuclear positivity but also a further decrease in cellularity. It is noteworthy that the cellular phenotype in the three zones revealed a fibro-chondrocytic pattern (Figure 3, arrows). In the matrix, organized immunopositive fibers were present in all ages.

Collagen type I outer part—In the outer part of the meniscus, the immunopositivity for collagen type I fibers was evident in all the considered zones and ages (Figure 3, arrowheads). In the outer part of the anterior horn, body, and posterior horn of the FD samples, the meniscal matrix showed a higher degree of organization of the fibers when compared to PD and ND.

Aggrecan inner part—Aggrecan revealed negative nuclear immunopositivity in the inner part of anterior horn, body and posterior horn in ND meniscus, and no evident fibers pattern was observed. PD and FD groups showed a nuclear immunopositivity (Figure 4, arrows) and a clear organization of the fibers (Figure 4, arrowheads), especially related to the body of the FD where the cells were located along the traction forces. Cellularity decreased during growth.

Aggrecan outer part—Aggrecan immunopositivity reflected the pattern of the inner part: A more organized fiber disposition in the matrix was evident, particularly in the FD samples (Figure 4, arrowheads).

### 3.4. Biomechanical Analysis: Compression and Traction Tests

We considered as representative value the Elastic Modulus (E) returned by each specimen at the third applied strain ramp, corresponding to 12% strain, both for compression and traction. 

Compression test—Results of the compression test are reported in Figure 5B, C. Whole FD menisci revealed statistically higher values when compared to ND and PD (*p* < 0.01, Figure 5B), and the same trend was observed when the menisci were analyzed considering the three zones: anterior horn, central body, and posterior horn (Figure 5C; *p* < 0.01 all comparisons, Figure 4).

Circumferential traction test—The Elastic modulus of the central body increased during growth in an age-dependent manner. When compared, FD and PD meniscal body showed a similar result, whereas ND showed a significantly lower value with respect to all comparisons (*p* < 0.01, Figure 5E). Moreover, the mean value of the Elastic modulus of the PD menisci showed significantly lower values compared to FD, both in anterior horn (*p* < 0.01) and posterior horn (*p* < 0.05), while no differences were observed in the central body (Figure 5F).

## 4. Discussion

Results obtained in the present study showed age-dependent changes in structural and biomechanical properties of porcine meniscal extracellular matrix. This variation clearly reflects the progressive maturation and hyper-specialization of the meniscus.

The GAGs/DNA ratio and a progressive change of meniscal cells from a fibrous to cartilaginous phenotype indicated an increasing production of matrix from ND to PD to FD, according to what was observed in bovine menisci [29]. The switch in cellular phenotype from fibroblast-like cells to mature fibro-chondrocytes was accompanied by a decrease in collagen I production [9,11]. Morphologically, ND/PD menisci expressed collagen I, which was homogeneously distributed in the matrix in the outer part of the anterior horn, in the body, and in the posterior horn. Moreover, an initial linear organization was noted only in the posterior horn, as previously described [30]. The fibroblastic phenotype in ND/PD menisci was probably due to an inconsistent compressive stimulus at this age, as also confirmed by the aggrecan distribution. Indeed, we observed an age-dependent increase deposition of aggrecan from ND to FD menisci; similar results were found in human menisci [31]. Moreover, cells in the central body of the PD meniscus were disposed along the tensile force lines, thus confirming the ability of fibroblast-like cells to differentiate when specific biomechanical stimuli are present [32]. On the contrary, cells of FD samples were reduced in number but showed an increased immunopositivity, volume, and a spherical shape in the inner zone, reflecting a meniscal fibrochondrocyte differentiation [23,30,32] and proving their mature phenotype, as previously reported [24]. This means that in the inner part of mature menisci, there were specialized cells secreting components of the extracellular matrix. Melrose et al. [11] deal with a comparative spatial and temporal localization of aggrecan and collagen I in the ovine meniscus from 2 days to 10 years old. This report shows that aggrecan is strongly immunolocalized to the tip of the inner zone. However, collagen I is uniformly immunolocalized throughout the outer middle and inner zones of the meniscus at all time points examined, confirming the fibrocartilaginous classification of this tissue type.

All these aspects were reflected in the mechanical properties of the different menisci. The Elastic modulus in compression (Ec) was comparable in ND and PD animals. These results indicated a small resistance to compressive forces, with a high grade of deformation of the meniscus when loaded. On the contrary, compression tests revealed that the FD meniscus greatly resists to deformation, due to the composition of the extracellular matrix, in particular the higher content of aggrecan. Indeed, this macromolecule possesses high osmotic properties, which are crucial to counteract compressive loads on the tissue [25,26].

Different results were obtained in traction: the PD/FD meniscal body showed an increased resistance compared to ND, whereas the anterior and posterior horns in PD showed a lesser resistance compared to FD, indicating that collagen I organization was not yet functional. All these considerations confirmed that the matrix maturation starts at this age in pigs (around 1 month), concomitantly with a cellular phenotype differentiation [30]. However, these features were still too immature to express a functional biomechanical behavior and to manifest an organized ultrastructure. The Elastic modulus in the FD meniscus was revealed to be elevated, and this indicates its ability to counteract tensile forces, mainly in the anterior horn. This property was dependent on collagen I organization in the matrix [33,34].

The mature extracellular matrix found in the FD meniscus conferred the strength properties, as shown by the biomechanical tests. Collagen I and aggrecan were slightly more expressed in the anterior horn and in the central body. Anterior horn is the most stressed part in normal stifle during locomotion, as well as the meniscal body. Higher differentiation of these parts of the meniscus is a consequence of stifle kinematics. Previous studies have reported that the anterior portion of the medial meniscus has the highest stiffness in different species, while the posterior region showed the lowest values [35]. These findings are also similar in human meniscus, where the anterior region showed the greater stiffness [36]. In our study, we also noted regional differences, but only in the traction test, where the anterior horn showed a greater stiffness. Considering the whole of the data, our results are consistent with the literature. The morphological changes in swine meniscus reflect different biomechanical behaviors: both the compression and tensile forces increase with age. Hyper-specialization of the meniscus is obtained by sacrificing its vascularization and regenerative capacity, as we have already observed in previous works of our group: we have observed that there is a clear correlation between cellular differentiation in a fibrocartilaginous tissue, thus leading to meniscal maturation with progressive reduction in blood supply [16]. Considering age-dependent characteristics, the PD category represents the transition phase, when these animals start to walk. Thus, locomotion is an essential stimulus to boost the meniscus maturation.

## 5. Conclusions

The present study showed age-dependent changes in structural and biomechanical properties of porcine meniscal extracellular matrix. This variation clearly reflects the progressive maturation and hyper-specialization of the meniscus.

Increasing evidence has revealed that a different array of additional environmental factors contributes to the overall control of stem cell activity, focusing on the importance of the extracellular matrix [37,38,39]. The extracellular matrix (ECM) is an “informational” entity as it receives, imparts and integrates structural and functional signals [40]. The mechanism of this “dynamic reciprocity” between cells and ECM is still partially unknown, but many studies have investigated the matrix [38,39,41,42,43]. Data on the important influence that the extracellular matrix has on cell fate are still growing. It is possible that the ECM-based control of the cell may also occur through multiple physical mechanisms, such as ECM geometry at the micro- and nanoscale, ECM elasticity, or mechanical signals [37].

These studies show that physical interactions with the ECM significantly influence the cell behavior, and that it can interact with chemical (i.e., composition), molecular (i.e., soluble mediators), or genetic (cell-type) factors in order to regulate cell fate.

A greater understanding of its structural characteristics can provide useful insights into the overall understanding of the development of the meniscus itself. Studies about meniscal maturation in animal models could represent landmarks for the development of artificial scaffolds. A new scaffold might be compared during all its creation processes with meniscal characteristics seen in nature, such as the prediction of the success of the product. These are future goals of research, and these will require cooperation between clinicians, biotechnologists, and researchers all from different scientific fields.

## Data Availability

Publicly available datasets were analyzed in this study. These data can be found here: https://osf.io/62xpm/?view_only=dc75d461115c4f669113948cd0094777 (accessed on 18 February 2022).
